# Structure characterization and immunoactivity on dendritic cells of two neutral polysaccharides from *Dictyophora rubrovalvata*

**DOI:** 10.1007/s13659-024-00476-6

**Published:** 2024-09-14

**Authors:** Ni Huang, Yi-Na Yang, Jia Huang, Hui-Yan Shao, Yan-Lang Li, Shi-Hui Qin, Han-Fen Li, Xiao-Jiang Shen, Liu Yang, Jiang-Miao Hu

**Affiliations:** 1 Key Laboratory of Phytochemistry and Natural Medicines, Kunming Institute of Botany, Chinese Academy of Sciences, Kunming, 650201 Yunnan China; 2 University of Chinese Academy of Sciences, Beijing, 100049 China; 3 College of Pharmacy, Anhui University of Chinese Medicine, Hefei, 230012 Anhui China

**Keywords:** *Dictyophora rubrovalvata*, Polysaccharides, Structure characterization, Immunoactivity

## Abstract

*Dictyophora rubrovalvata* is a valuable fungus homologous to food and medicine, and its polysaccharide have been gaining increasing attention because of its plentiful activity. However, the structure and activity of its homogeneous polysaccharide have not been studied enough. In this study, two polysaccharides DRP-I and DRP-II were purified from *D. rubrovalvata*. Their structures were characterized by chemical composition, monosaccharide composition analysis, methylation analysis and nuclear magnetic resonance spectroscopy. The results showed that DRP-I and DRP-II were neutral heteropolysaccharides with molecular weights of 5.79 × 10^3^ and 1.25 × 10^4^ Da, respectively, which were composed of mannose, galactose, glucose, xylose and fucose. The main chains were → 6)-*α*-D-Gal*p*-(1 → 6)-*α*-D-Gal*p*-(2,1 → 6)-*α*-D-Man*p*-(2,1 → 6)-*α*-D-Gal*p*-(1, and branch chains were *β*-D-Xyl*p*-(1 → 3)-*α*-L-Fuc*p*-(1 → 4)-*α*-D-Man*p*-(1 → and *α*-D-Gal*p*-(1 → 3)-*α*-D-Gal*p*-(1 → . The in vitro immunoactivity assays on dendritic cells showed that DRP-I and DRP-II could up-regulate the expression of IL-10 and IL-6 and inhibit the expression of TNF-α in a concentration-dependent manner. This research indicated that DRP-I and DRP-II possessed immunoactivity by balancing the excessive inflammation, and molecular weight is an important factor affecting immunoactivity.

**Graphical Abstract:**

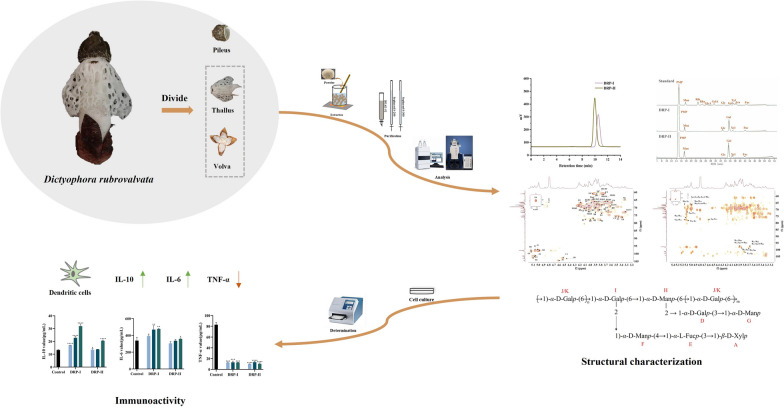

**Supplementary Information:**

The online version contains supplementary material available at 10.1007/s13659-024-00476-6.

## Introduction

Edible mushroom polysaccharides are important natural active macromolecules, which have attracted extensive attention due to their unique structures and abundant biological activities [1]. It has been reported that most of polysaccharides in edible mushroom are composed by D-glucan, and there are also heteropolysaccharides such as mannose, arabinose and galactose [2, 3]. And exhibiting a variety of biological activities such as immunomodulatory, anti-inflammatory, hypoglycemic, and anti-tumor [4–7]. Immunoactivity is one of the most important biological activities of edible mushroom polysaccharides, they can exert immunoactivities by influencing the expression of cytokines, activating MAPK and other signaling pathways, and indirectly acting on intestinal flora [8]. Structure–activity relationship studies have shown that the activity of polysaccharides is affected by polymerization degree (molecular weight), monosaccharide composition, type of glycosidic bond, branching degree and higher conformation [9].

*Dictyophora rubrovalvata* is one of the traditional edible and medicinal mushrooms belonging to *Dictyophora* genus, Phallaceae, Basidiomycota, which is identified by professor Zang Mu and cultivated wildly in China nowadays [10]. Its mature fruiting body includes thallus (consists of stipe and indusium), pileus, and volva [11]. Previous researches showed that polysaccharides were the mainly active components of *D. rubrovalvata*, and they exhibited satisfactory immunoenhancing, anti-tumor, antihyperlipidemic and antioxidant effects [12–15]. These indicated that *D. rubrovalvata* was an important source of active polysaccharide. However, the structures and activities of homogeneous polysaccharides still need further study.

In this study, two neutral polysaccharides (DRP-I and DRP-II) have been purified from thallus and volva of *D. rubrovalvata*. Meanwhile, their structures were characterized by molecular weight, chemical compositions, monosaccharide compositions, methylation analysis and UV, IR and NMR spectroscopy. In addition, the in vitro immunoactivity was evaluated using dendritic cells, including cell proliferation capacity and cytokine (IL-10, IL-6 and TNF-α) secretion capacity.

## Results and discussion

### Isolation and purification

**Fig. 1:**
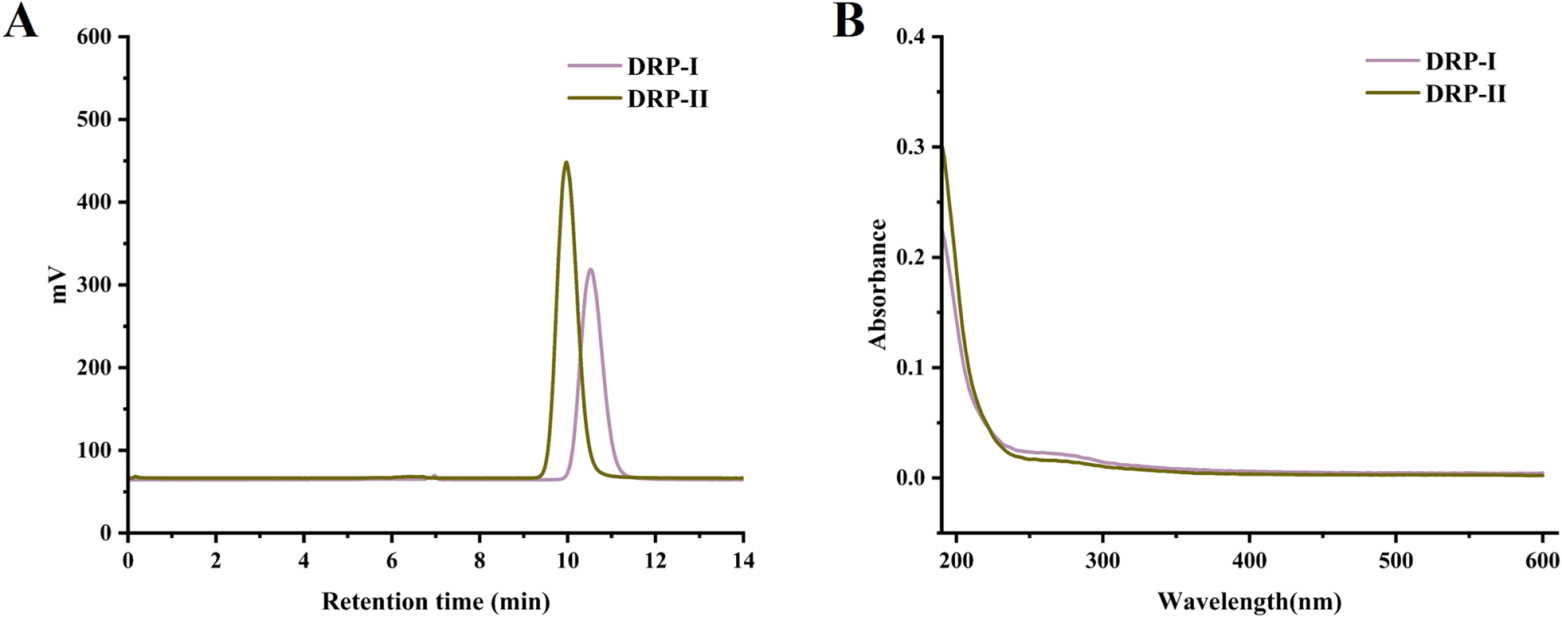
High-performance gel permeation chromatography profiles of DRP-I and DRP-II (**A**). The UV spectra of DRP-I and DRP-II (**B**)

Through the hot water extraction, alcohol precipitation and purification by ion exchange column and gel column, two neutral polysaccharides named DRP-I and DRP-II were obtained, respectively. As shown in Fig. 1A, they exhibited single and symmetrical sample peaks analyzed by high-performance gel permeation chromatography (HPGPC), which indicated that they were homogeneous polysaccharides.

### Molecular weight and chemical compositions determination

The molecular weights of DRP-I and DRP-II were determined using HPGPC. The contents of total sugar and protein of them were determined by the phenol–sulfuric acid method and Bicinchoninic-acid method. As shown in Table 1, the molecular weight of DRP-I and DRP-II were 5.79 × 10^3^ Da and 1.25 × 10^4^ Da, and the total sugar contents were 92.78% and 88.43%, respectively. The results of UV spectrum (Fig. 1B) and protein detection assay (Table 1) pointed out DRP-I and DRP-II contained almost no other impurities.

**Table 1:** The molecular weight, chemical and monosaccharide compositions of DRP-I and DRP-II

Parameters	DRP-I	DRP-II
Molecular weight (Da)	5.79 × 10^3^	1.25 × 10^4^
Total sugar content (W%)	92.78	88.43
Protein content (W%)	0.82	0.14
Monosaccharide composition		
Mannose	17.04	20.04
Glucose	9.62	2.47
Galactose	60.80	57.90
Xylose	6.48	11.18
Fucose	6.06	6.20

### Monosaccharide composition analysis

**Fig. 2:**
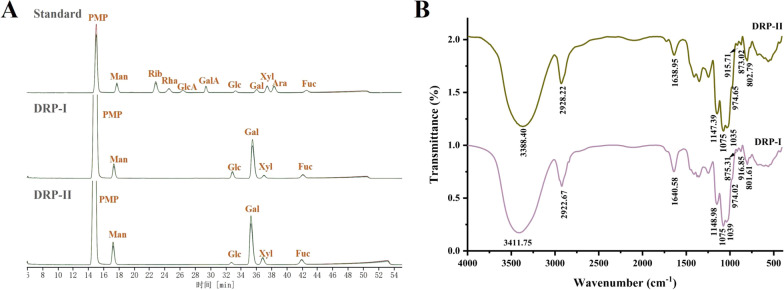
The HPLC profiles of monosaccharide analysis of DRP-I and DRP-II (**A**). The FT-IR spectra of DRP-I and DRP-II (**B**)

The monosaccharide compositions of DRP-I and DRP-II were shown in Fig. 2A, DRP-I was composed of mannose (17.04%), glucose (9.62%), galactose (60.80%), xylose (6.48%) and Fucose (6.06%), and DRP-II was composed of mannose (20.04%), glucose (2.47%), galactose (57.90%), xylose (11.18%) and Fucose (6.20%). They had the same monosaccharide composition and no significant difference in proportion.

### Infrared spectral analysis

The FT-IR spectrums of DRP-I and DRP-II were shown in Fig. 2B, they both presented the characteristic peaks of polysaccharide. The strong and wide absorptions at approximately 3300-3400 cm^−1^ attributed to the O–H stretching vibration, the peaks at about 2925 cm^−1^ were the stretching vibration of C-H from CH, CH_2_ and CH_3_, the absorption peaks at about 1640 cm^−1^ were caused by water, and the C-O stretching vibration and O–H bending vibration appeared in approximately 1460–1200 cm^−1^ [16]. In addition, the three absorption peaks at 1200–1000 cm^−1^ are C–O–C stretching vibration of pyranoid sugar ring [17].

### Methylation analysis

DRP-I and DRP-II were methylated, acetylated, GC–MS analyzed and compared the *m/z* results of PMAAs with the Complex Carbohydrate Research Center database (https://www.ccrc.uga.edu) to obtain linking information. The results were presented in Table 2, Fig. S1, and Fig. S2, indicating that DRP-I and DRP-II were complex polysaccharides with 10 linkage fragments.

**Table 2:** The glycosidic linkages of DRP-I and DRP-II determined by methylation analysis

t_*R*_/min	Methylated sugar	Mass fragments (*m/z*)	Type of linkage
7.720	2,3,4-Me_3_-Xyl*p*	59,73,87,101,102,117,118,161,162	T-Xyl*p*
11.107	2,4-Me_2_-Fuc*p*	59,72,89,101,113,118,131,160,234,243	→ 3)-Fuc*p*-(1 →
11.711	2,3,4,6-Me_4_-Glc*p*/Man*p*	43,71,87,102,118,129,145,162,205	T-Glc*p*/Man*p*
12.379	2,3,4,6-Me_4_-Gal*p*	43,71,87,102,118,129,145,162,205	T-Gal*p*
15.082	2,4,6-Me_3_-Gal*p*	59,74,87,101,118,129,161,174,202,234,277	→ 3)-Gal*p*-(1 →
15.370	2,3,6-Me_3_-Man*p*	59,71,87,102,118,129,143,162,173,233	→ 4)-Man*p*-(1 →
17.235	2,3,4-Me_3_-Gal*p*	59,71,87,99,118,129,162,173,189,233	→ 6)-Gal*p*-(1 →
20.857	3,4-Me_3_-Gal*p*	74,87,99,100,129,130,189,190,233	→ 2,6)-Gal*p*-(1 →
20.980	3,4-Me_3_-Man*p*	43,87,101,118,129,139,160,189,234	→ 2,6)-Man*p*-(1 →

### NMR analysis

NMR analysis was used to further elucidate the structures of DRP-I and DRP-II. In generally, the polysaccharide’s chemical shifts of anomeric hydrogen and anomeric hydrogen of the *α*-configuration are *δ*_H_ 5.1–5.8 ppm and *δ*_C_ 98–103 ppm, while the corresponding chemical shifts of the *β*-configuration are *δ*_H_ 4.3–4.8 ppm and *δ*_C_ 103–106 ppm [18]. The ^1^H, ^13^C and HSQC spectra of DRP-I showed that there were 11 kinds of anomeric hydrogen and carbon signal peaks of the sugar residue segments (Fig. 4 and Table 3), and 4.61/104.43, 4.46/103.32, 4.53/102.97, 5.11/102.40, 5.09/101.67, 5.07/101.47, 5.13/98.20, 5.14/98.30, 5.06/98.00, 5.01/97.86 and 4.99/97.81 ppm signals in HSQC spectra were labeled as A, B, C, D, E, F, G, H, I, J and K, respectively. According to ^1^H-^1^H COSY and TOCSY  spectra, 4.61/3.32, 4.61/3.45, 4.61/3.63 and 4.61/3.96 ppm signals indicate that there were correlations between A_H1_ subsequent corresponding protons A_H2-5_. Therefore, the signals 3.32/73.02, 3.45/75.65, 3.63/69.20 and 3.96;3.33/65.06 ppm in the HSQC spectrum were assigned to A_H2/C2_ ~ A_H5/C5_ of residue A, indicating that residue A was *β*-Xyl*p*-(1 → through methylation results and comparison with earlier literature [19]. The 3.58/70.95 and 3.69/72.54 ppm signals in the HSQC spectrum were assigned to residue *β*-D-Gal*p*-(1 → (residue B) [20]. Similarly, residue C could be inferred to *β*-D-Glc*p*-(1 →  [21]. And the cross peaks at 5.11/102.40, 4.00/77.57 and 3.75;3.89/61.02 ppm in the HSQC spectrum were attributed to D_H1/C1_, D_H3/C3_ and D_H6/C6_ of residue D, low-field chemical shift at C3 (77.57 ppm) of residue D indicating it was → 3)-*α*-D-Gal*p*-(1 →  [22]. The signals at 5.09/101.6, 4.08/77.57 and 1.23/15.19 ppm in the HSQC spectrum were attributed to E_H1/C1_, E_H3/C3_ and E_H6/C6_ of residue E, and signal 1.23/15.19 ppm belonged to methyl and low-field shift at C3 (77.57 ppm) suggesting residue E was → 3)-*α*-L-Fuc*p*-(1 →  [23]. The residues F was assigned to → 4)-*α*-D-Man*p*-(1 → , because of the signals 5.07/101.47 and 3.84/71.69 ppm [24]. Residue G could be identified as *α*-D-Man*p*-(1 →  [25]. Residue H was confirmed to be 2,6)-*α*-D-Man*p*-(1 → according to the signals 5.14/98.30 and 3.94/76.95 ppm in the HSQC spectrum and methylation analysis results [25]. And the residue I was assigned to → 2,6)-*α*-D-Gal*p*-(1 → due to the signals at 5.06/97.99, 3.82/78.05 and 3.87;3.72/66.44 ppm in the HSQC spectrum [26]. Finally, residues J and K were identified as → 6)-*α*-D-Gal*p*-(1 → based on the chemical shifts of their anomeric carbons and chemical shifts of C6 moving towards lower field [27].

**Table 3:** The ^1^H and ^13^C chemical shifts of DRP-I

Residues	H1/C1	H2/C2	H3/C3	H4/C4	H5/C5	H6/C6
A	4.61/104.43	3.32/73.02	3.45/75.65	3.63/69.20	3.96,3.33/65.06	
B	4.46/103.32	3.58/70.95	3.69/72.54	–	–	–
C	4.53/102.97	3.32/73.02	3.49/75.55	3.46/69.20	3.63/69.22	3.72,3.83/60.70
D	5.11/102.40	4.08/69.78	4.00/77.57	3.90/72.54	4.16/68.78	3.75,3.89/61.02
E	5.09/101.67	3.90/70.37	4.08/77.57	3.77/73.35	4.18/67.19	1.23/15.19
F	5.07/101.47	3.79/68.80	3.90/68.25	3.84/71.69	–	–
G	5.13/98.20	4.11/70.37	3.66/72.87	3.60/66.75	3.38/76.22	3.75,3.93/61.02
H	5.14/98.30	3.94/76.95	4.02/70.99	–	–	3.87,3.72/66.44
I	5.06/97.99	3.82/78.05	4.06/68.25	3.97/67.49	3.85/68.25	3.87,3.72/66.44
J	5.02/97.86	3.84/68.25	4.02/69.46	3.89/68.21	4.20/68.78	3.91,3.68/66.44
K	5.00/97.81	3.84/68.25	4.02/69.46	3.89/68.21	4.20/68.78	3.91,3.68/66.44

**Fig. 3:**
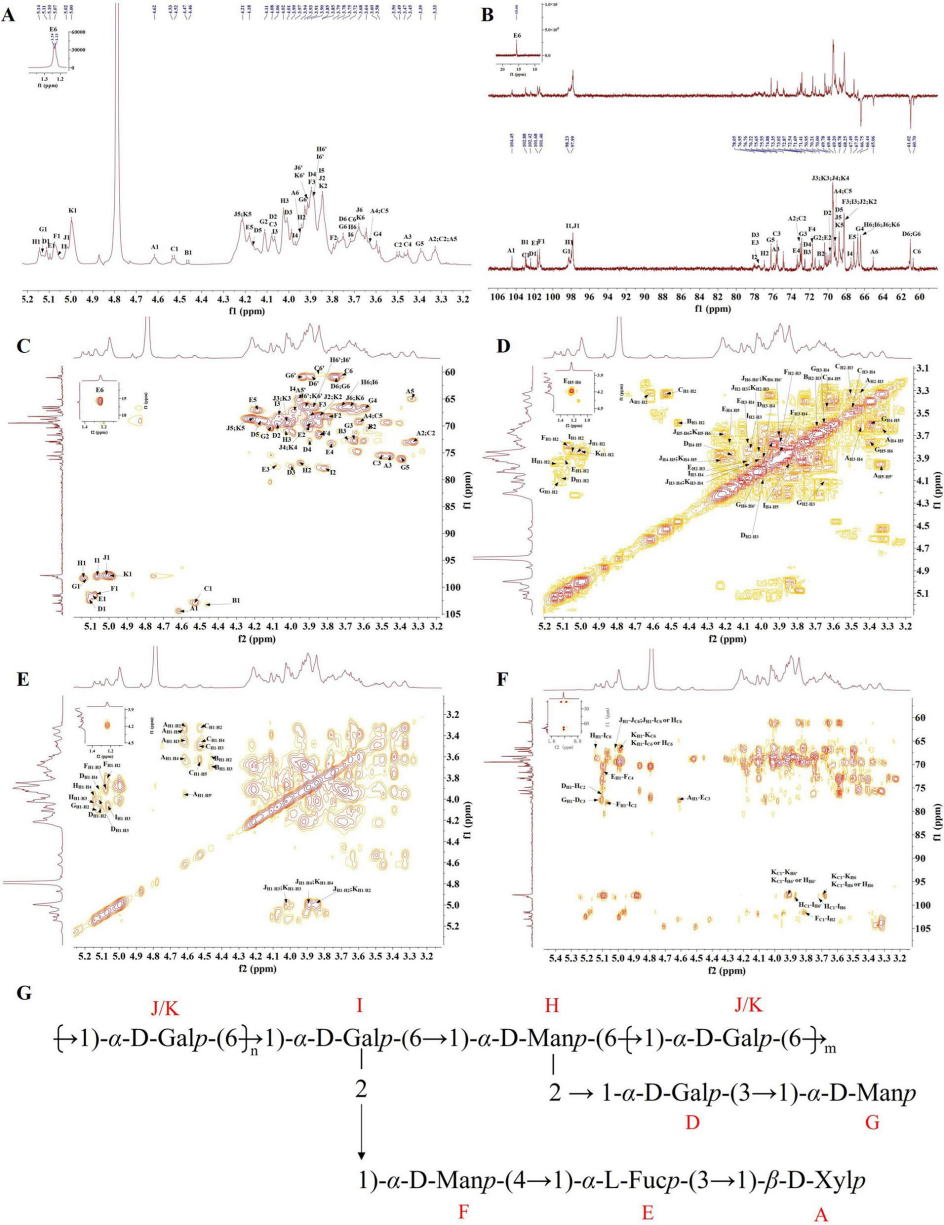
The ^1^H (**A**), ^13^C and DEPT-135(**B**), HSQC (**C**), ^1^H-^1^H COSY (**D**), TOCSY (**E**), and HMBC(**F**) spectra of DRP-I. The proposed repeated unit of DRP-I (**G**)

The proposed repeated unit of DRP-I was analyzed by the HMBC spectrum. The cross peaks of A_H1_-E_C3_ (4.61/77.57 ppm), E_H1_-F_C4_ (5.09/71.69 ppm) and F_H1_-I_C2_ (5.071/78.05 ppm) indicated the presence of sequence of *β*-D-Xyl*p*-(1 → 3)-*α*-L-Fuc*p*-(1 → 4)-*α*-D-Man*p*-(1 → 2)-Gal*p*-(1,6 → ; and cross peaks of G_H1_-D_C3_ (5.13/77.57 ppm), D_H1_-H_C2_ (5.11/76.95 ppm) suggested the side chain of *α*-D-Gal*p*-(1 → 3)-*α*-D-Gal*p*-(1 → 2)-Man*p*-(1,6 → . Besides, the cross peaks of *δ*_C_ 66.44 ppm in the HMBC spectrum were attributed to → 6)-*α*-D-Gal*p*-(1 → 6)-*α*-D-Gal*p*-(2,1 → 6)-*α*-D-Man*p*-(2,1 → 6)-*α*-D-Gal*p*-(1. Therefore, combined with the results of monosaccharide composition analysis, methylation analysis and NMR analysis, DRP-I was the branched neutral heteropolysaccharide with the proposed repeated unit derived in Fig. 3G. Moreover, DRP-II had similar monosaccharide composition, linkage fragments, and NMR spectrums with DRP-I, suggesting the similarity of their structures (Fig. S3 and S4). Comparing with the ultrasonic assisted water-extracted polysaccharides from pileus of *D. rubrovolvata* previously, DRP-I and DRP-II have different molecular weight, monosaccharide composition and linkages, suggesting that the structural features of polysaccharides may depend on extraction part and method [15].

### SEM analysis

**Fig. 4:**
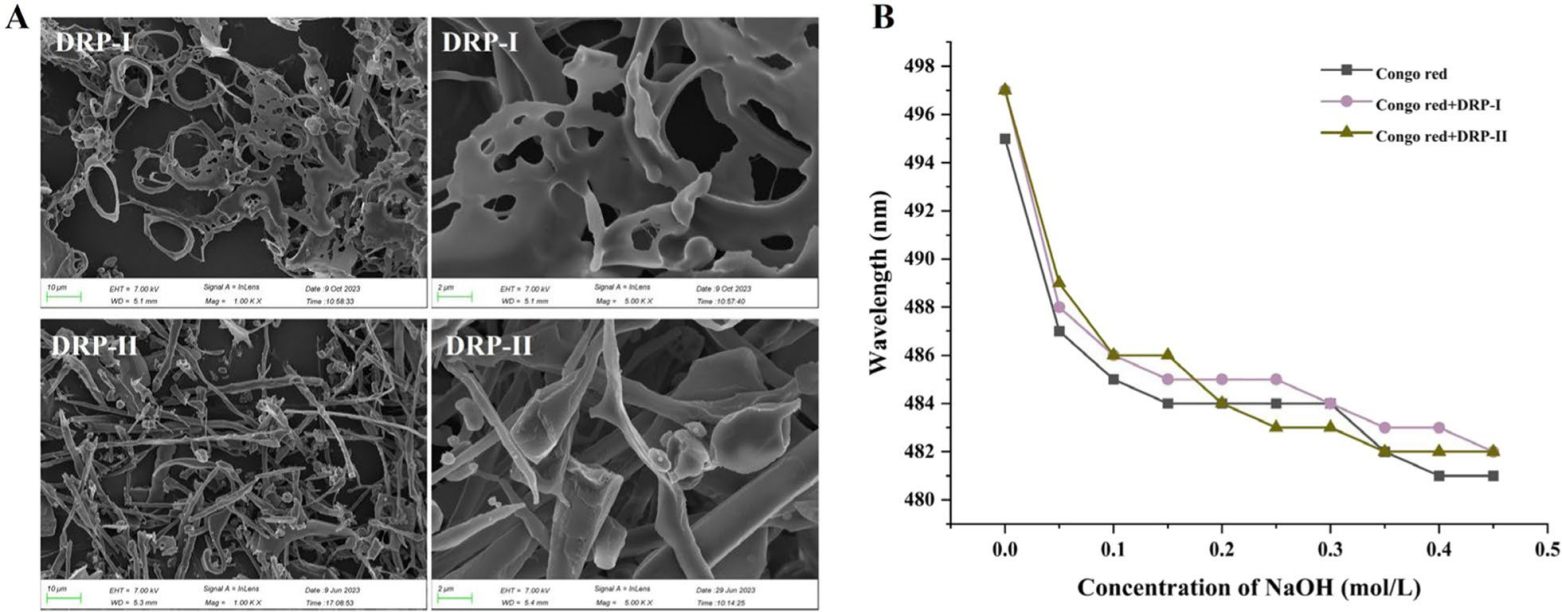
The SEM detection result of DRP-I and DRP-II (**A**). The maximum absorption wavelength of Congo red and DRP-I and DRP-II at various concentrations of sodium hydroxide solution (**B**)

Scanning Electron Microscope (SEM) detection results of DRP-I and DRP-II (Fig. 4A) showed that the microscopic apparent form of DRP-I is lamellar with pores, while DRP-II is rod-like, spheroidal and lamellar form. DRP-I exhibits a more orderly apparent morphology.

### Congo red analysis

When the Congo red and triple-helix conformation polysaccharide form the complex at a range of low base concentration, its maximum absorption wavelength (λmax) moves towards a long wavelength, so that the triple-helix conformation of polysaccharide can be identified by Congo red experiment [28]. The λ_max_ of congo red and DRP-I ~ DRP-II + congo red at different NaOH concentrations  were shown in Fig. 4B. At 0.00–0.20 M NaOH, *λ*max decreased first and then tended to be stable. These results indicated that DRP-I and DRP-II did not have triple-helix conformation.

### Immunoactivity on dendritic cells

**Fig. 5:**
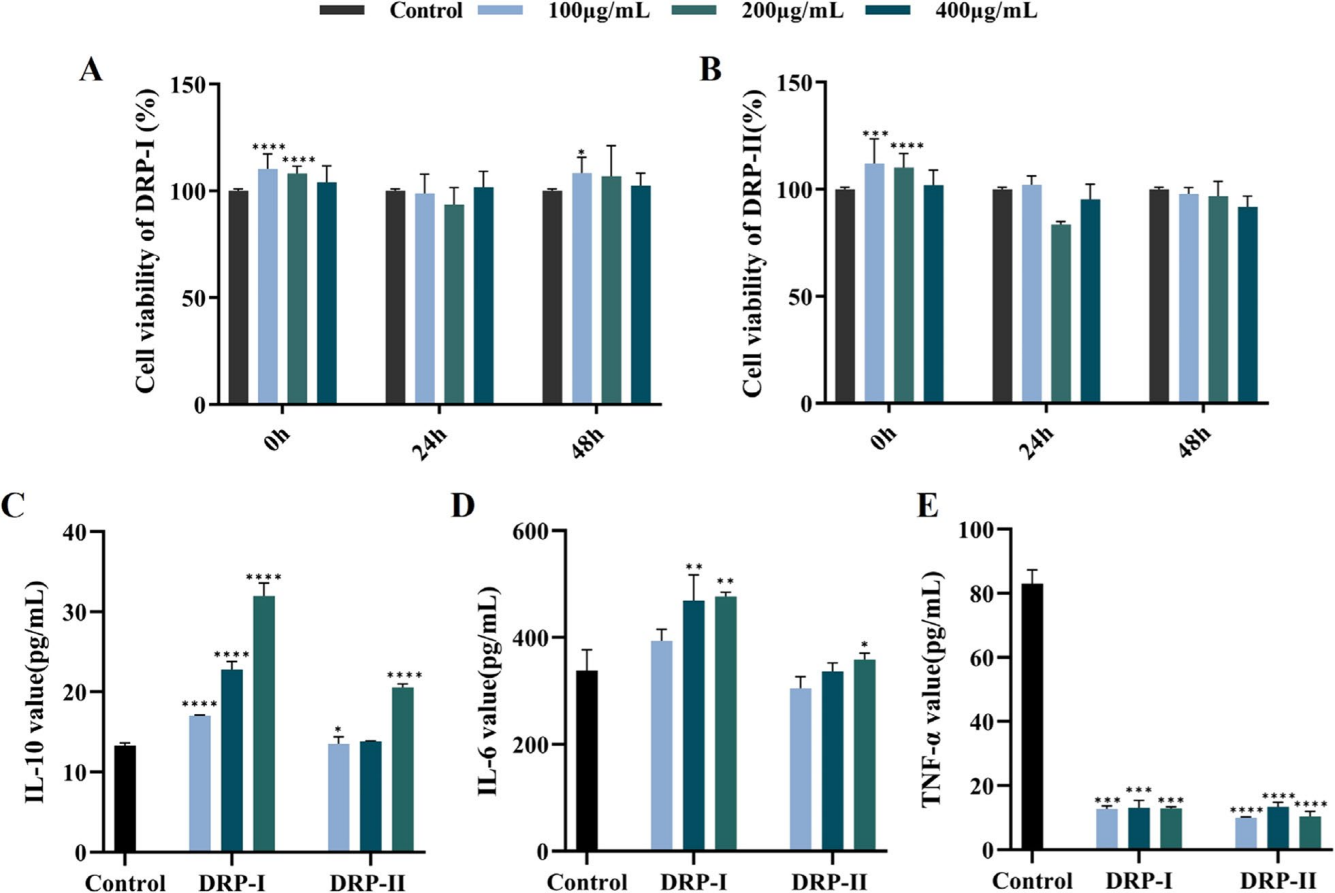
The immunomodulatory activity of DRP-I and DRP-II on dendritic cells. The proliferation on dendritic cells of DRP-I (**A**) and DRP-II (**B**). The effects of DRP-I and DRP-II on IL-10 (**C**), IL-6 (**D**) and TNF-α (**E**) secretion on dendritic cells. *0.01 < *p* ≤ 0.05, **0.001 > *p* ≤ 0.01, ***0.0001 < *p* ≤ 0.001, ****p < 0.0001 vs Control Group

The immunoactivities of DRP-I and DRP-II were evaluated using dendritic cells. The effect on proliferation was determined by MTS method, as shown in Fig. 5A and B, DRP-I and DRP-II showed no cytotoxicity at concentrations ranging of 100–400 μg/mL. The effects of DRP-I and DRP-II on cytokines (Fig. 5C, D and E) showed that they could promote the secretion of the anti-inflammatory cytokine IL-10 and the pleiotropic cytokine IL-6 and inhibit the secretion of the pro-inflammatory cytokine TNF-α with dose-dependent manner, and the immunoactivity of DRP-I was more significant. It might be suggested that DRP-I and DRP-II balancing the excessive inflammation to perform immunoactivity [29]. Based on the receptor-active center theory, polysaccharides with lower polymerization degree are more likely to bind to the active receptor center, which may be the reason for the more significant activity of DRP-I [30].

## Discussion and conclusion

In this work, two polysaccharides named as DRP-I and DRP-II were purified from *D. rubrovalvata*, with molecular weights of 5.79 × 10^3^ and 1.25 × 10^4^ Da, respectively. They were composed of mannose, glucose, galactose, xylose and fucose. And the main chains  were → 6)-*α*-D-Gal*p*-(1 → 6)-*α*-D-Gal*p*-(2,1 → 6)-*α*-D-Man*p*-(2,1 → 6)-*α*-D-Gal*p*-(1, the branch chains were *β*-D-Xyl*p*-(1 → 3)-*α*-L-Fuc*p*-(1 → 4)-*α*-D-Man*p*-(1 → and *α*-D-Gal*p*-(1 → 3)-*α*-D-Gal*p*-(1 → . DRP-I and DRP-II showed immunoactivity by up-regulating the expression of IL-10 and IL-6 and inhibiting the expression of TNF-α on dendritic cells, in which DRP-I had more significant immunoactivity. In summary, DRP-I and DRP-II are potential natural immunomodulators, and the molecular weight of polysaccharides is closely related to immunomodulatory activity, which lays a foundation for the development of immunomodulatory polysaccharides and the study of their structure–activity relationship.

## Experimental section

### Chemicals and reagents

The fresh *D. rubrovalvata* was obtained from Songming, Kunming, China in July 2022.The samples were identified by Zhao Qi, a senior engineer at the Kunming Institute of Botany, Chinese Academy of Sciences.

Standard molecular weight dextrans (1, 5, 12, 25, 470, 610 kDa) were purchased from Sigma-Aldrich Co., Ltd (Shanghai, China). DEAE-52 was purchased from Solarbio Science & Technology Co., Ltd (Beijing, China). Sephacryl S-200 and S-300 were purchased from Cytiva Bio-technology Co., Ltd (Hangzhou, China). Arabinose, fucose, galactose, galacturonic acid, glucose, glucuronic acid, mannose, rhamnose, xylose and ribose were purchased from Aladdin Co., Ltd (Shanghai, China). 1-phenyl-3-methyl-5-pyrazolone (PMP) were purchased from Adamas Reagent Co., Ltd. (Shanghai, China). Methyl iodide was purchased from TCI Development Co., Ltd. (Shanghai, China). Deuteroxide (D_2_O) was purchased from Adamas Reagent Co., Ltd. (Shanghai, China). Congo red was purchased from Sigma-Aldrich Co., Ltd (Shanghai, China). RPMI-1640 medium was purchased from BI Co., Ltd. (Shanghai, China). The mouse IL-10 ELISA kit, mouse IL-6 ELISA kit and mouse TNF-*α* ELISA kit were purchased from Fine Biotech Co., Ltd. (Wuhan, China).

### Extraction and purification

The fresh fruiting bodies of *D. rubrovalvata* were divided into volva, thallus and pileus. They were soaked with 95% ethanol to remove pigments and other alcohol-soluble compounds (3 times, each time for 12 h), and ventilated at room temperature to dry, respectively. The dried thallus of *D. rubrovalvata* were crushed and extracted with hot water (w: v = 1:20, 3 times, each time for 2 h). The water extract was collected by centrifuging, and then concentrated. The protein of water extract was removed by the Sevage method (chloromethane: n-butanol = 4:1, v/v). Subsequently, the water extract was mixed with anhydrous ethanol for the final ethanol concentration to reach 60% (v/v) to precipitate polysaccharides. The precipitates were re-dissolved in hot distilled water and removed the residual ethanol. At last, the crude thallus and volva polysaccharides of *D. rubrovalvata* (DRTP60 and DRVP60) were obtained by lyophilization.

The crude polysaccharides (DRTP60 and DRVP60, 10 mg/mL) were subjected on DEAE-52, which were eluted with distilled water, 0.1 M NaCl, 0.3 M NaCl and 0.5 M NaCl solution at a flow rate of 2.0 mL/min. And the concentrated eluents were further purified on the Sephacryl S-200 and Sephacryl S-300 gel permeation columns. The columns were eluted with distilled water at a flow rate of 0.5 mL/min. The eluents were tested by by HPLC system (Agilent, USA) equipped with the evaporative light scattering detector (ELSD, Alltech, USA) and Shodex KS-804 column (7.8 mm × 300 mm) and the same fractions were combined. And then the purified and single fractions (DRP-I and DRP-II) were obtained by lyophilization.

### Structure characterization of DRP-I and DRP-II

#### Chemical compositions

The total sugar content was measured with phenol–sulfuric acid method [31]. The protein content was determined by Bicinchoninic-acid method [32]. And the UV spectrophotometer was used to detect the presence of proteins and nucleic acids that have ultraviolet absorption by scanning in the range of 190–600 nm.

#### Molecular weight and homogeneity determination

The average molecular weight and homogeneity of DRP-I and DRP-II were determined by HPLC-ELSD system equipped with Shodex KS-804 column (7.8 mm × 300 mm). The standard dextrans with different molecular weights (1, 5, 12, 410, 670 kDa) were used to establish a standard molecular weight curve.

#### Monosaccharide composition analysis

The analytical method of monosaccharide compositions of DRP-I and DRP-II referred to Dai et al. with Simple modification [33]. 2.0 mg of polysaccharides DRP-I and DRP-II were hydrolyzed with 4 mol/L TFA at 90 °C for 8 h, and the residual TFA was removed by adding methanol repeatedly and drying under pressure. The hydrolyzed sample and monosaccharide standard (arabinose, fucose, galactose, galacturonic acid, glucose, glucuronic acid, mannose, rhamnose, xylose and ribose) were derivatized by 1-phenyl-3-methyl-5-pyrazolone (PMP). They were prepared into the solutions of 1 mg/mL and mixed with 50 μL of NaOH (0.6 M) and 100 μL of PMP (0.5 M), respectively. The reaction was performed at 70 ℃ for 100 min. After the reaction and cooling to room temperature, the mixtures were added 100 μL of HCl (0.3 M), 1 mL distilled water and 1 mL chloroform. The supernatant was extracted after chloroform extraction (3 times) and filtered for HPLC analysis.

#### Infrared (IR) spectral analysis

Approximately 1 mg of dried polysaccharides of DRP-I and DRP-II evenly mixed with KBr powder, tableted. And then they were analyzed with FT-IR spectroscopy (Bruker, Germany) in the range of wave length 4000–500 cm^−1^.

#### Methylation analysis

The analytical method of methylation of DRP-I and DRP-II referred to Ciucan, Kerek and Liang et al. with Simple modification [34, 35]. 5.0 mg of polysaccharides DRP-I and DRP-II were dissolved with anhydrous DMSO completely, filled with N_2_ gas, and then NaOH (20 mg) was added. After ultrasonic mixed for 20 min, the mixture was cooled and solidified. Then dropping CH_3_I (1.5 mL) slowly and ultrasonic reacted for 30 min. The methylation reactions were terminated by adding 1 mL distilled water. Repeat the above steps until the methylation was complete. The methylated polysaccharides were hydrolyzed with 4 mol/L TFA at 110 °C for 4 h. Subsequently, NaBD_4_ (10 mg/mL) was added to reduce samples, and acetic anhydride / anhydrous pyridine (v/v = 1:1) were added to acetylate (120 ℃, 120 min). The acetylation products were extracted by CH_2_Cl_2_ for GC–MS analysis.

#### NMR analysis

The freeze-dried polysaccharides of DRP-I and DRP-II (10 mg) were dissolved in D_2_O (0.5 mL). Repeatedly freeze-dried to replace the H in the polysaccharides with D. The 1D and 2D NMR data were measured by Bruker Advance 800 MHz NMR spectrometer (Bruker, Germany).

#### SEM analysis

The freeze-dried polysaccharides of DRP-I and DRP-II attached on the conductive adhesive and gold-plated. And then the samples were observed their surface morphology at different magnifications by scanning electron microscope (Carl Zeiss, Germany).

#### Congo red analysis

Congo red assay was used to analyze whether the polysaccharide had a triple-helix conformation [36], and the steps were as follows: In the experimental group, polysaccharide solution (2 mg/mL) was mixed with Congo red solution (80 μM) in equal volume (50 μL), and then 100 μL NaOH solution with different concentrations was added respectively. The final concentrations of NaOH in the mixed solution were 0.00, 0.05, 0.10, 0.15, 0.20, 0.25, 0.30, 0.35, 0.40 and 0.45 M, respectively. After incubating at 25 ℃ for 10 min, the UV absorption of the samples in the range of 400–700 nm was analyzed by microplate reader. The control group was deionized water mixed with Congo red solution in equal volume, and the specific steps were the same as above.

### Immunoactivity on dendritic cells

#### Cells culture

Bone marrow derived dendritic cells (BMDCs) were cultured in RPMI-1640 medium (containing 10% FBS and 1% double antibody) at 37 ℃ and 5% CO2.

#### Proliferation assay

The proliferation of DRP-I and DRP-II were determined by MTS method on BMDCs [4]. 100 μL of BMDCs (1 × 10^5^ cell/mL) were cultured in 96-well cell plates, after the cells were attached to the wall, the supernatant was discarded. The experimental group was added with DRP-I and DRP-II (100, 200 and 400 μg/mL, 200 μL), and the blank control group was added with RPMI-1640 base medium (200 μL). The culture was continued for different time (24 h and 48 h). Then the supernatant of cells in the 96-well plate was discarded, and MTS solution (20 μL) and RPMI-1640 base medium (100 μL) were added to each well, and cultured under the same conditions for 1 h. The absorbance of each well was measured at 490 nm by microplate reader.

#### IL-10, IL-6 and TNF-α determination

The effects of DRP-I and DRP-II on cytokines (IL-10, IL-6 and TNF-α) secreted on BMDCs were determined by ELISA kits [4]. The density of BMDCs was adjusted to 1 × 10^5^ cell/mL. 100 μL of BMDCs (1 × 10^5^ cell/mL) were cultured in 96-well cell plates, after the cells were attached to the wall, the supernatant was discarded. The experimental group was added with DRP-I and DRP-II (100, 200 and 400 μg/mL, 200 μL), and the blank control group was added with RPMI-1640 base medium (200 μL). And the supernatant was collected after culture for 24 h. Then the concentrations of IL-6, IL-10 and TNF-α were determined according to the ELISA kit instructions, respectively.

### Statistical analysis

Data were expressed as the mean ± SD (standard deviation) of triplicate determinations. Statistical significance was analyzed by one-way analysis of variance (ANOVA) and GraphPad Prism software (GraphPad, San Diego, CA, USA).  *p*< 0.05 was considered statistically significant.

## Supplementary Information

Supplementary Material 1. Additional file SI:  Fig. S1: The methylation GC-MS results of DRP-I. Fig. S2: The methylation GC-MS results of DRP-II. Fig. S3. The ^1^H NMR and ^13^C NMR spectra of DRP-I and DRP-II. Fig. S4 The ^1^H (A), ^13^C and DEPT-135(B), HSQC (C), COSY (D), TOCSY (E), and HMBC(F) spectra of DRP-II.

## Data Availability

The datasets used or analyzed during the current study are available from the corresponding author on reasonable request.
