# Supplementary material for: Structure characterization and immunoactivity on dendritic cells of two neutral polysaccharides from Dictyophora rubrovalvata
Source: Nat Prod Bioprospect. 2024 Sep 14;14(1):52. doi: 10.1007/s13659-024-00476-6 (PMC11401800; doi:10.1007/s13659-024-00476-6)
Supplement: Supplementary file 1 — Supplementary Material 1. Additional file SI: Fig. S1: The methylation GC-MS results of DRP-I. Fig. S2: The methylation GC-MS results of DRP-II. Fig. S3. The 1H NMR and 13C NMR spectra of DRP-I and DRP-II. Fig. S4 The 1H (A), 13C and DEPT-135(B), HSQC (C), COSY (D), TOCSY (E), and HMBC(F) spectra of DRP-II. [file 13659_2024_476_MOESM1_ESM.docx]

**Structure characterization and immunoactivity on dendritic cells of two neutral polysaccharides from *Dictyophora rubrovalvata***

**Supplementary materials**


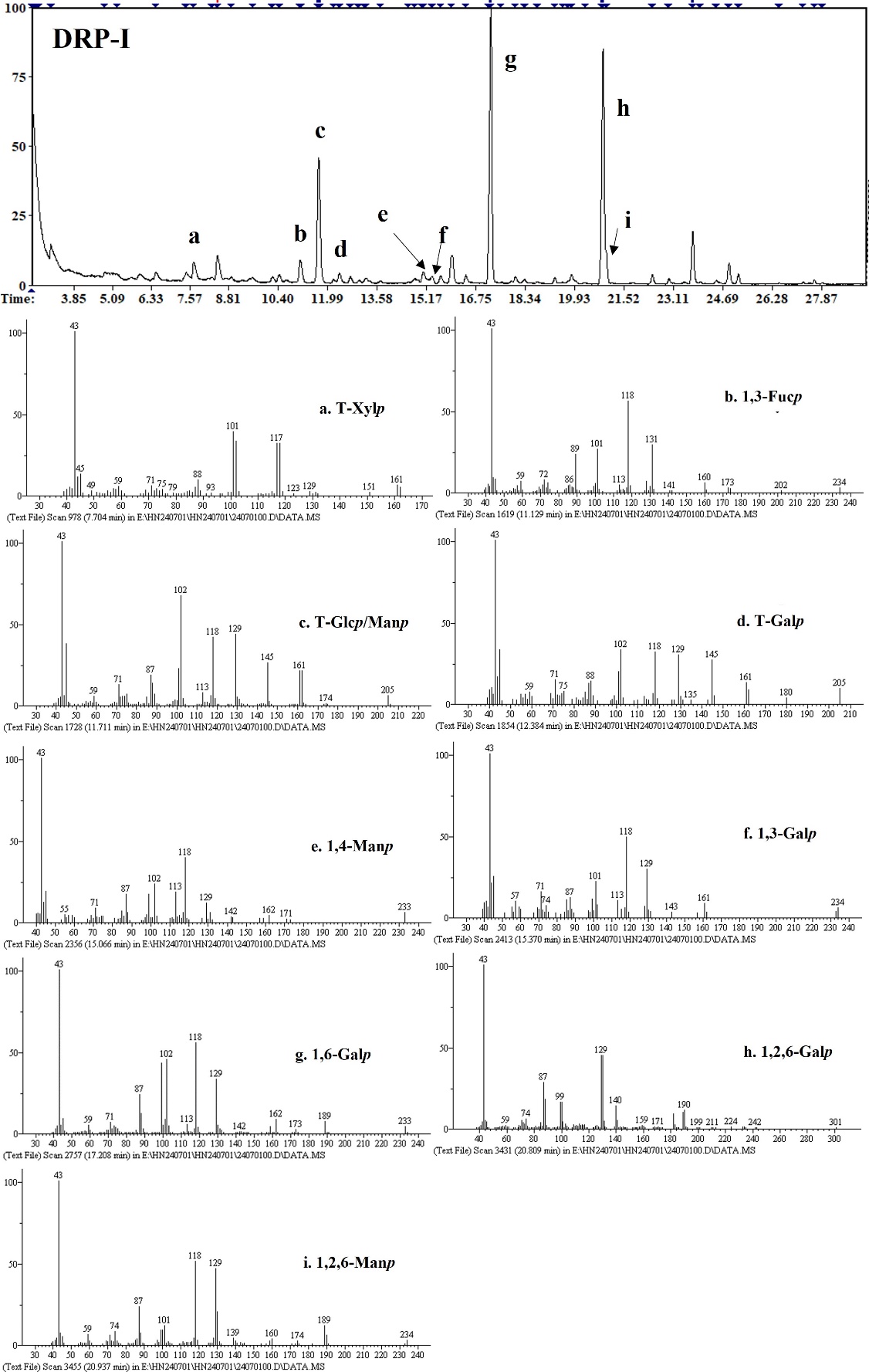


Fig. S1 The methylation GC-MS results of DRP-I.


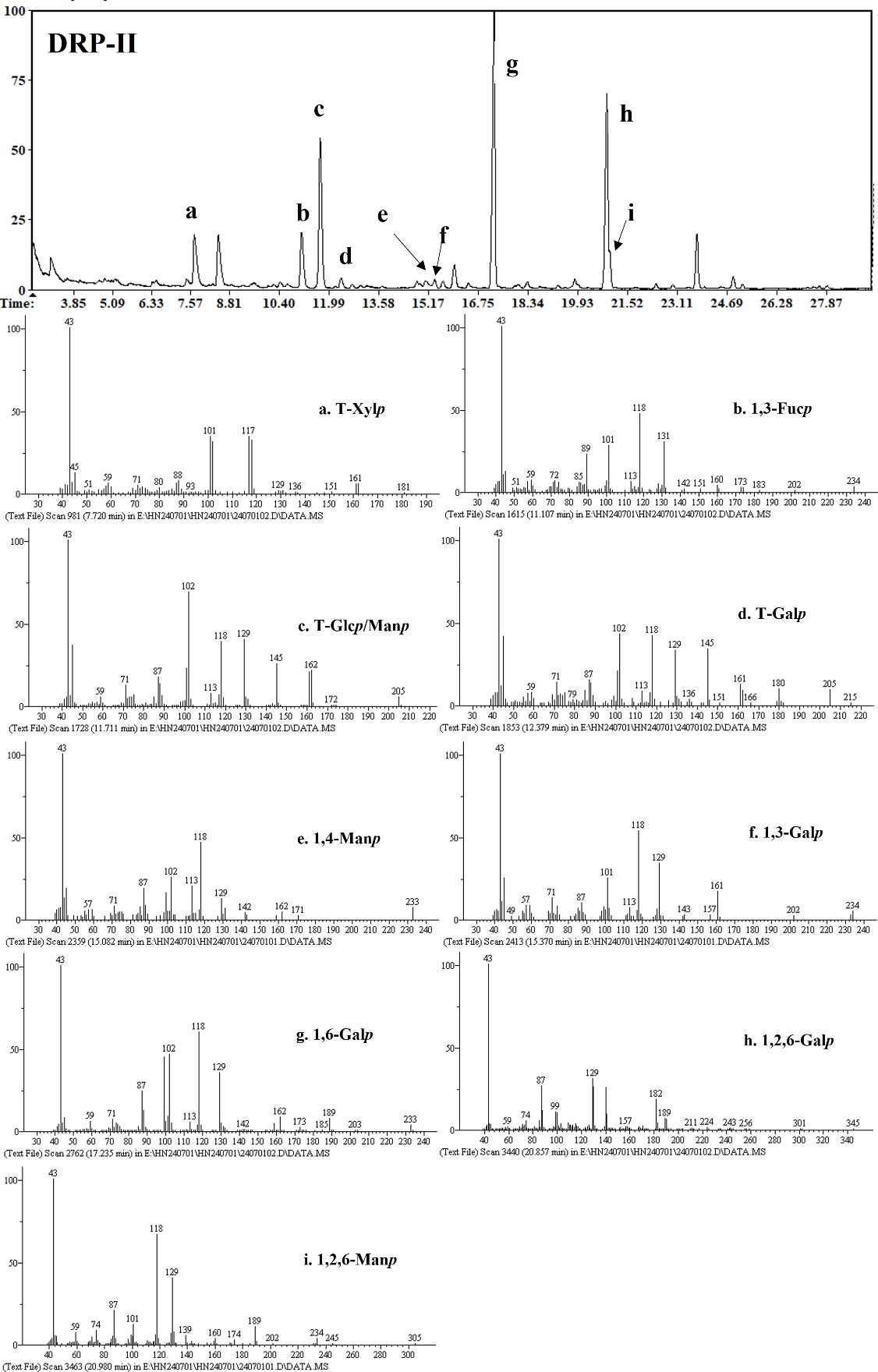


Fig. S2 The methylation GC-MS results of DRP-II.


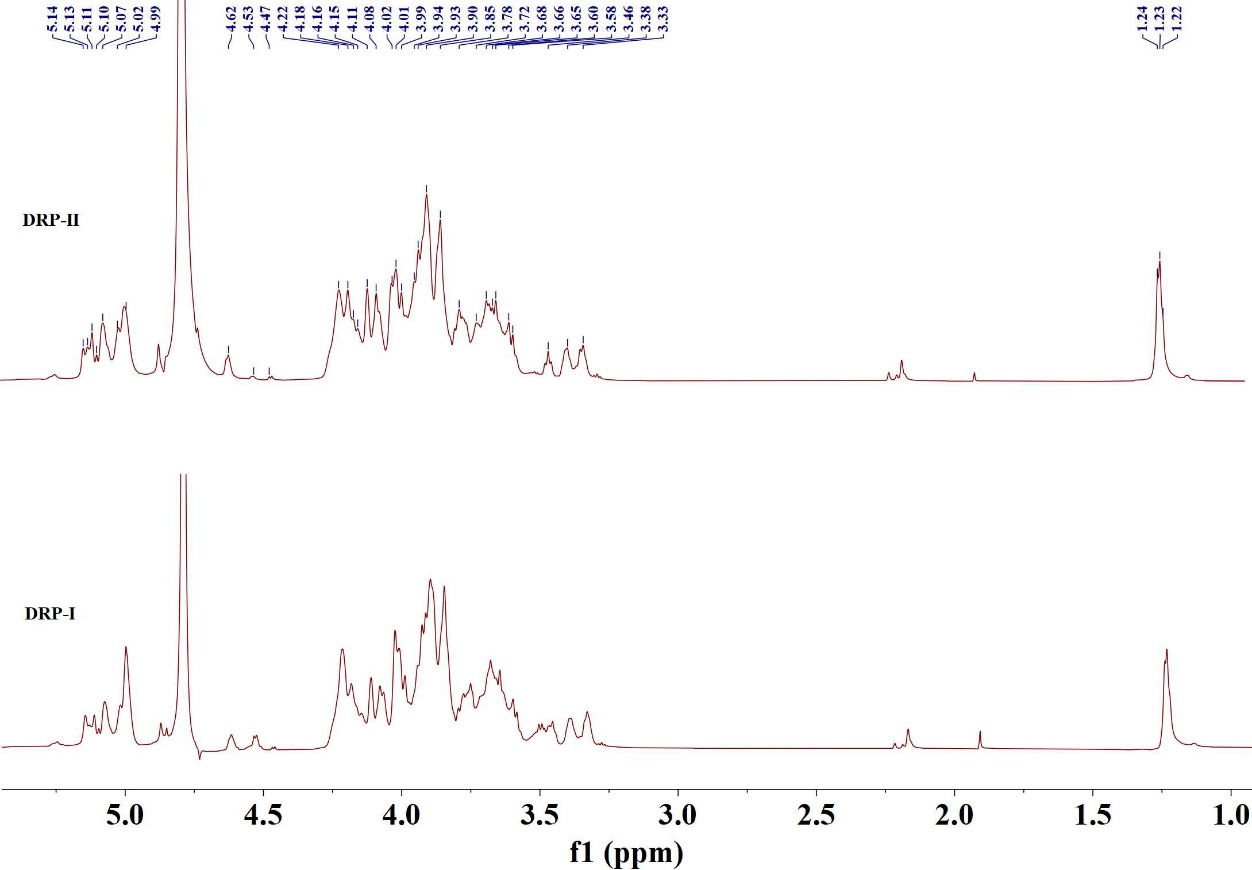

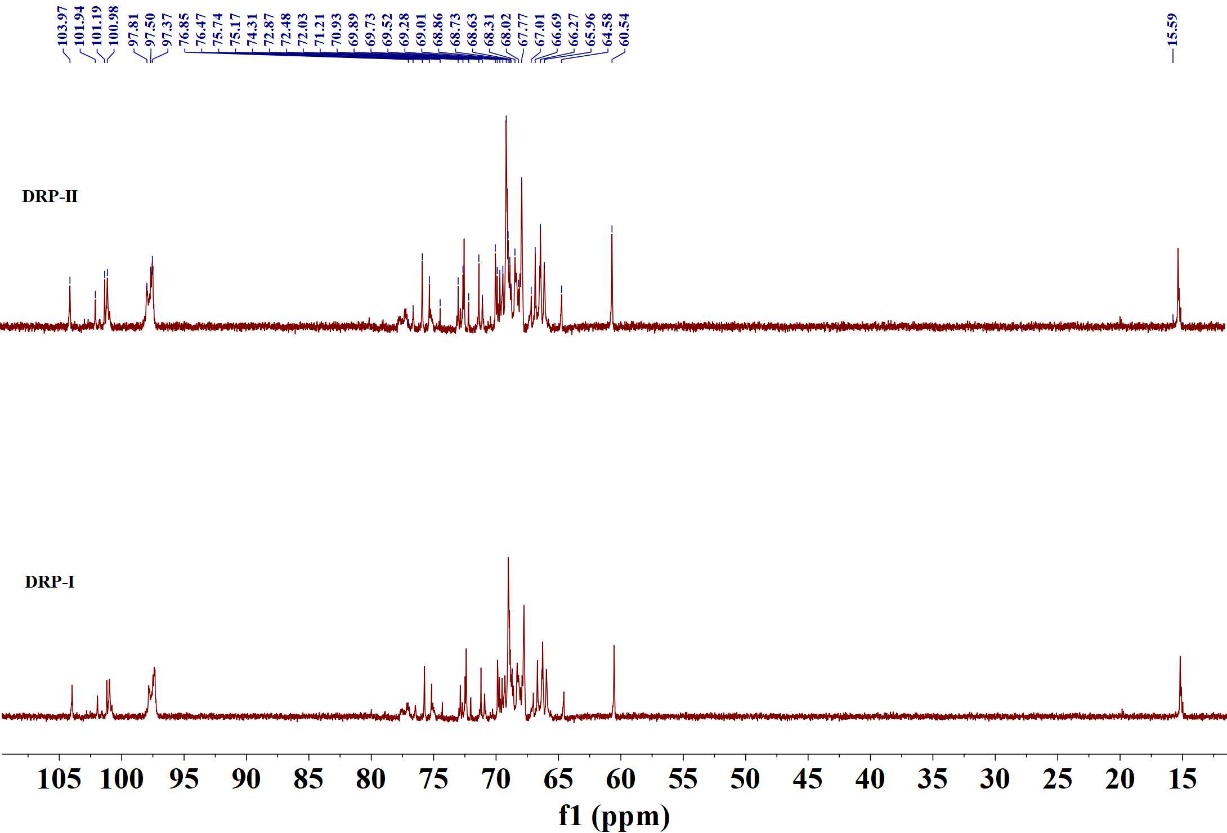


Fig. S3 The ^1^H NMR spectra and ^13^C NMR spectra of DRP-I and DRP-II.


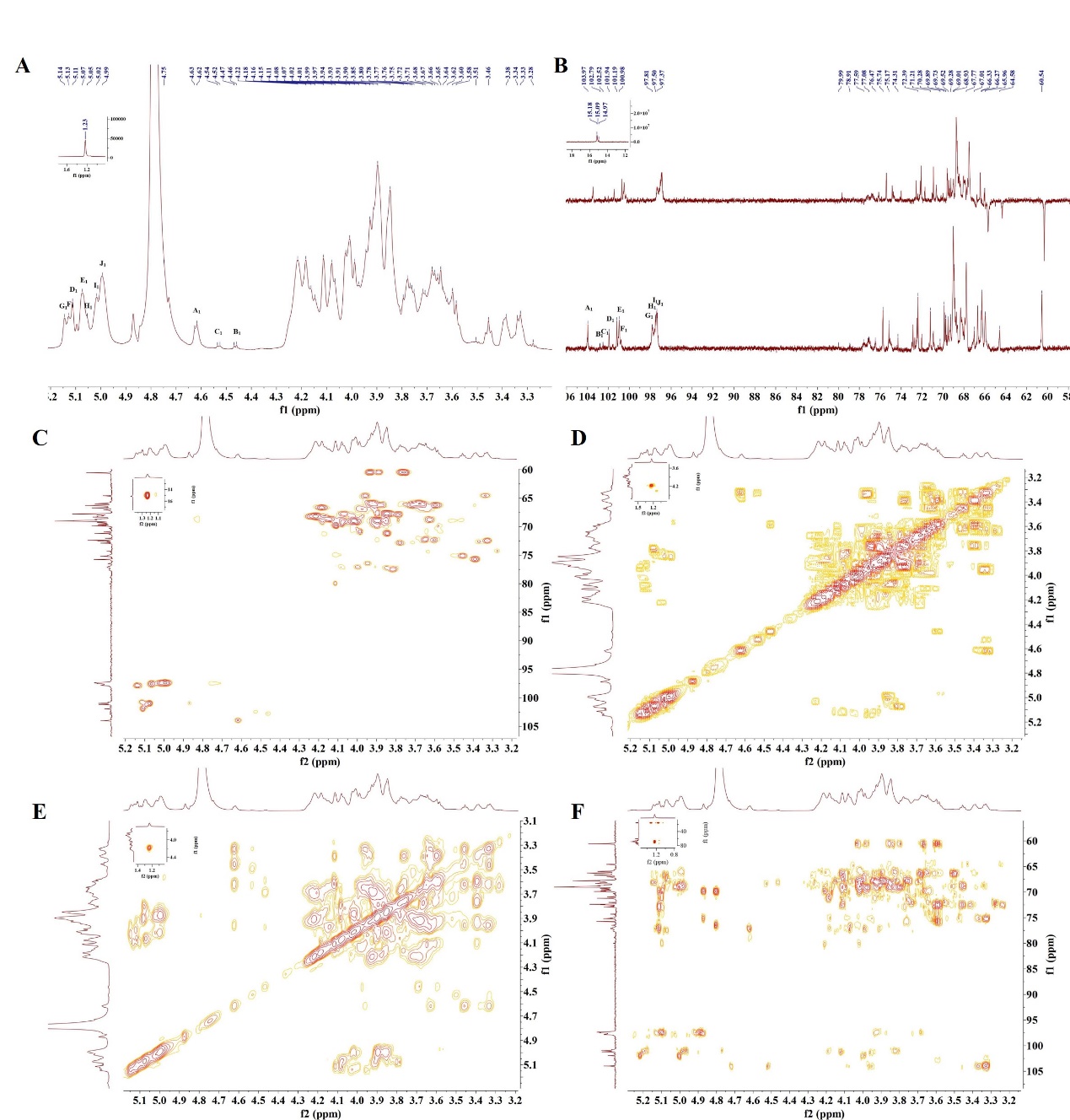


Fig. S4 The 1H (A), 13C and DEPT-135(B), HSQC (C), COSY (D), TOCSY (E), and HMBC(F) spectra of DRP-II.
